# *Aedesaegypti*: egg morphology and embryonic development

**DOI:** 10.1186/s13071-021-05024-6

**Published:** 2021-10-13

**Authors:** Ana Paula Miranda Mundim-Pombo, Hianka Jasmyne Costa de Carvalho, Rafaela Rodrigues Ribeiro, Marisol León, Durvanei Augusto Maria, Maria Angélica Miglino

**Affiliations:** 1 University Nilton Lins, Manaus, Amazonas Brazil; 2 Present Address: Department of Surgery, Anatomy Sector, Faculty of Veterinary Medicine and Animal Science, University of São Paulo, Avenida Professor Orlando Marques de Paiva, 87, Butantã, São Paulo, São Paulo CEP 05508-270 Brazil; 3 Molecular Biology Laboratory of the Butantan Institute, São Paulo, Brazil

**Keywords:** Eggs of *Aedes aegypti*, Public health, Arboviruses, Entomology ultrastructural analysis

## Abstract

**Background:**

The diseases for which *Aedes aegypti* is a vector are worrisome. The high vector competence of this mosquito, as well as its anthropophilia and ability to adapt to the urban environment, allows it to exploit many habitats, making its prevention an arduous task. Despite current disease control measures focused on the mosquito, the effectiveness in containing its dispersion still requires improvement; thus greater knowledge about this insect is fundamental.

**Methods:**

*Aedes aegypti* egg morphology and embryonic development were analyzed from eggs of the insectary of the Institute of Biomedical Sciences of the University of São Paulo. Optical (light and confocal) and electronic (transmission and scanning) microscopy were used to analyze the morphological and ultrastructural features of the eggs. Embryos were observed in the initial (0–20.5 h after egg-laying), intermediate (20.6–40.1 h after egg-laying), and final (40.2–61.6 h) stages of development, and kept at a temperature of 28 °C ± 1 °C until collection for processing.

**Results:**

Eggs of *Ae. aegypti* were whitish at the time of oviposition, and then quickly became black. The egg length was 581.45 ± 39.73 μm and the width was 175.36 ± 11.59. Access to the embryo was difficult due to the egg morphology, point of embryonic development, and difficult permeability of the exochorion (mainly in fixation). Only about 5% of the collected eggs were successfully processed. In the initial stage of embryonic development, characteristics suggestive of intense cellular activity were found. In the intermediate stage, the beginning of the segmentation process was evident. In the final phase, it was possible to differentiate the cephalic region and the thoracic and abdominal segments.

**Conclusion:**

The chorion was found to be an important protective barrier and a limiting factor for the evaluation of the embryos and mosquito embryonic cells, indicating that further studies need to be carried out to identify the reason that this occurs.

**Graphical abstract:**

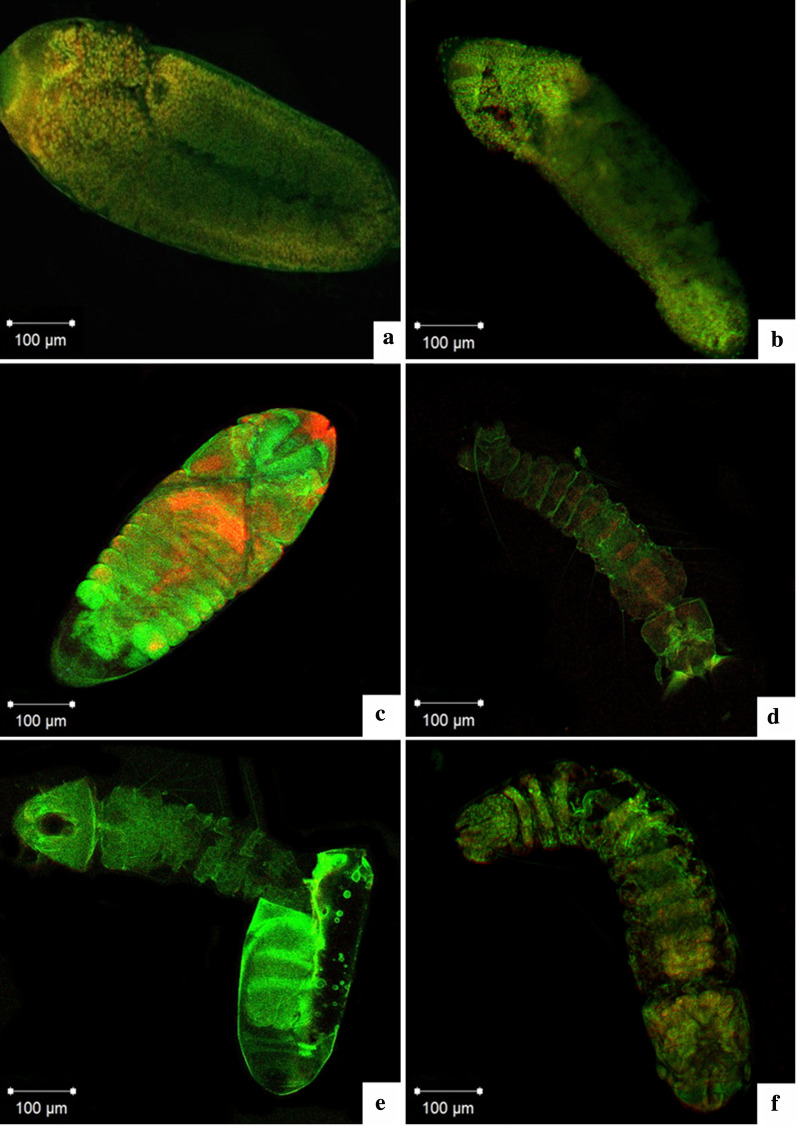

## Background

The mosquito *Aedes aegypti* is of considerable public health importance worldwide, mainly due to its involvement in the transmission of arboviruses (arthropod-borne viruses). Diseases commonly related to this transmission are dengue [1, 2], chikungunya [3], and Zika [4–6]. Zika virus infection was reported for the first time in Brazil in May 2015 [7], and the epidemiological scenario involving the disease is characterized by the simultaneous movement of its etiological agents to various locations. This situation increases the risk of dispersion and the possibility of co-infection [8], factors that when correlated show the relevance of *Ae. aegypti* as a vector [7].

The magnitude of disease caused by the mosquito is of great concern. Factors involved include the pathogenicity of the infectious agents and the high competence of its main vector (*Ae. aegypti*), in addition to its anthropophilia and good adaptation to the urban environment, the context of climate change, and the process of accelerated urbanization, substandard housing policies, and urban infrastructure. These aspects can promote a favorable ambiance to the mosquito life cycle [9, 10] and act as obstacles to prevention measures and effective control with available resources [7, 11].

Moreover, effective vaccines for most arboviruses are lacking; for example, the first vaccine for dengue was licensed in 2015, but its performance is dependent on the patient’s serologic status [6, 12]. The World Health Organization recommends the administration of the vaccine only to seropositive individuals [13]. For chikungunya and Zika, there are several scientific obstacles to vaccine formulation, linked to the fragile balance between immunogenicity, safety, and the development of a safe vaccine for fetal life [14]. Thus it is evident that there is a need for high levels of vector-directed control [7, 9].

*Aedes aegypti* is a holometabolous insect and its biological cycle involves the egg, larval (four stages), pupal, and adult stages [15]. The focus of this study, the eggs, are particularly important, as they are resistant to dessication and can remain viable for approximately 1 year [15, 16], thus presenting a major obstacle to the control of *Ae. aegypti* [9, 16, 17]. The egg’s outer shell structure is called the chorion, which has a protective function and gas exchange function, and also minimizes water loss. This structure is composed of two distinct layers: the endochorion and exochorion [18]. The exochorion generally shows distinctive ornamentation, which is therefore an excellent marker for distinguishing significant differences between species [19–26].

*Drosophila melanogaster* has traditionally been the model used for the study of embryonic development in insects [27]. The challenges in describing the embryonic morphology of *Ae. aegypti* have been attributed to the egg permeability, which hinders the study of embryonic development [16], and is the reason embryogenesis is still a poorly understood part of the *Ae. aegypti* life cycle [28, 29]. Given the above and knowing that the early life cycle of the vector *Ae. aegypti* is the egg, an enhanced understanding of the morphological state and morphometric and embryonic developmental characteristics of *Ae. aegypti* is critical.

## Methods

### Obtaining biological material

*Aedes aegypti* eggs were donated by the Parasitology Department of the Institute of Biomedical Sciences of the University of São Paulo (USP), and were collected between November 2014 and February 2015. Forty-six eggs were analyzed in the study, even though about 1000 eggs were initially collected. For embryonic development analysis, a synchronization of egg-laying was performed by offering a container with water for oviposition for 30 min. After this period, the eggs were collected at three different times, namely the initial stage of embryogenesis (immediately after up to 20.5 h after egg-laying), the intermediate stage (20.6 to 40.1 h), and the final stage (40.2 to 61.6 h) of development, and kept at a temperature of 28 °C. Embryogenesis is completed in approximately 61.6 h when the temperature is 28 °C ± 1 °C, according to a previous study [25]. In each group of collected samples, development was interrupted when the eggs were subjected to the tissue fixation procedure. The morphological analyses were performed using optical microscopy (light and laser scanning confocal) and electronic microscopy (transmission and scanning).

### Optical microscopy: light microscopy

Samples were fixed in a 10% formaldehyde solution. After complete fixation, they were dehydrated in a series of increasing concentrations of ethanol (70 to 100%) and diaphonized in xylol, and were subsequent embedded in histological paraffin. Three-micrometer-thick cuts were made in the microtome (Leica, Germany) and stained with hematoxylin and eosin. Images were obtained using a Nikon Eclipse E800 light microscope at the Advanced Diagnostic Imaging Center/Faculty of Veterinary Medicine and Animal Science (FMVZ)-USP.

### Optical microscopy: laser scanning confocal microscopy

The eggs were initially processed by washing with water, followed by immersion in 3% sodium hypochlorite until clarification (approximately 30 min), with subsequent washing in phosphate-buffered saline (PBS) with 0.02% Triton for 5 min, and again in PBS. The material was then fixed in 3.7% formaldehyde for 20 min. After this procedure, a new washing sequence was performed with PBS, permeabilization using 1% Triton at room temperature and washing with PBS twice. The incubation was carried out in a dark room for 60 min, and fluorescein isothiocyanate (FITC)-labeled phalloidin was used. RNAse was added in the final 30 min of incubation. After this treatment, the embryos were again washed with PBS, and the nuclei were stained with propidium iodide. Fluorescent images were obtained by laser scanning confocal microscopy (Zeiss LSM 510) at the Cellular and Molecular Biology laboratory (BioCeM) of the Institute of Biomedical Sciences-USP.

### Transmission electron microscopy

The samples were fixed in 2% glutaraldehyde, and post-fixed in a 1% osmium tetroxide solution at 4° C and a 5% aqueous solution of uranyl acetate at room temperature. Then the samples were dehydrated in increasing concentrations of ethanol, immersed in propylene oxide, and soaked in Spurr resin. For light microscopy, semi-thin sections were cut using a Reichert Ultracut ultramicrotome and stained with 1% toluidine blue solution. Thin 90-nm sections were cut and collected in 200 mesh (Sigma-Aldrich) and contrasted with a 4% uranyl acetate solution and a 0.4% aqueous solution of lead citrate. The grids were examined using a transmission electron microscope at the Advanced Center for Diagnostic Imaging, FMVZ-USP. Specifically for the analysis of the semi-fine cut, the eggs were subjected to the same processing as described above, with washing in water, followed by immersion in sodium hypochlorite 3% until its clarification (approximately 30 min), and subsequent washing in PBS with 0.02% Triton for 5 min and again washed in PBS, except that they were then fixed in glutaraldehyde and the processing was continued.

### Scanning electron microscopy

The biological material was fixed in a modified Karnovsky solution (5% glutaraldehyde and 4% paraformaldehyde in 0.1 M cacodylate buffer, pH 7.2), followed by washing in sodium cacodylate buffer, pH 7.2, and posterior fixation in tetroxide osmium (OsO_4_) at 1% in 0.2 M sodium cacodylate buffer. After carrying out a new series of washing and cleaning controls, the eggs were dehydrated in an increasing series of ethanol to absolute ethanol (50%, 75%, 90%, and 100%). After passing a critical point, the material was mounted on stubs using double-sided carbon adhesive tape followed by sputter-coating with gold, and analyzed using scanning electron microscopy (SEM–AL FEI Quanta 250) in the Cell Biology Laboratory of the Butantan Institute.

### Data analysis

#### Morphometric analysis

Morphometric analysis of the eggs was performed with the images obtained by SEM. The following linear dimensions of the eggs were considered for this analysis:

• Length: distance between the micropyle and the opposite end
• Width: largest distance perpendicular to the length
• Egg index: length-to-width ratio
• Diameter of the micropylar disc: diameter of the anterior structure in the egg.

### Statistical analysis

After obtaining the measurements, analysis of the central tendency (mean) and measures of dispersion (standard deviation, maximum, and minimum values) was performed. Statistical analyses were carried out using GraphPad Prism software, considering a 95% confidence interval.

## Results

### Eggs

**Fig. 1:**
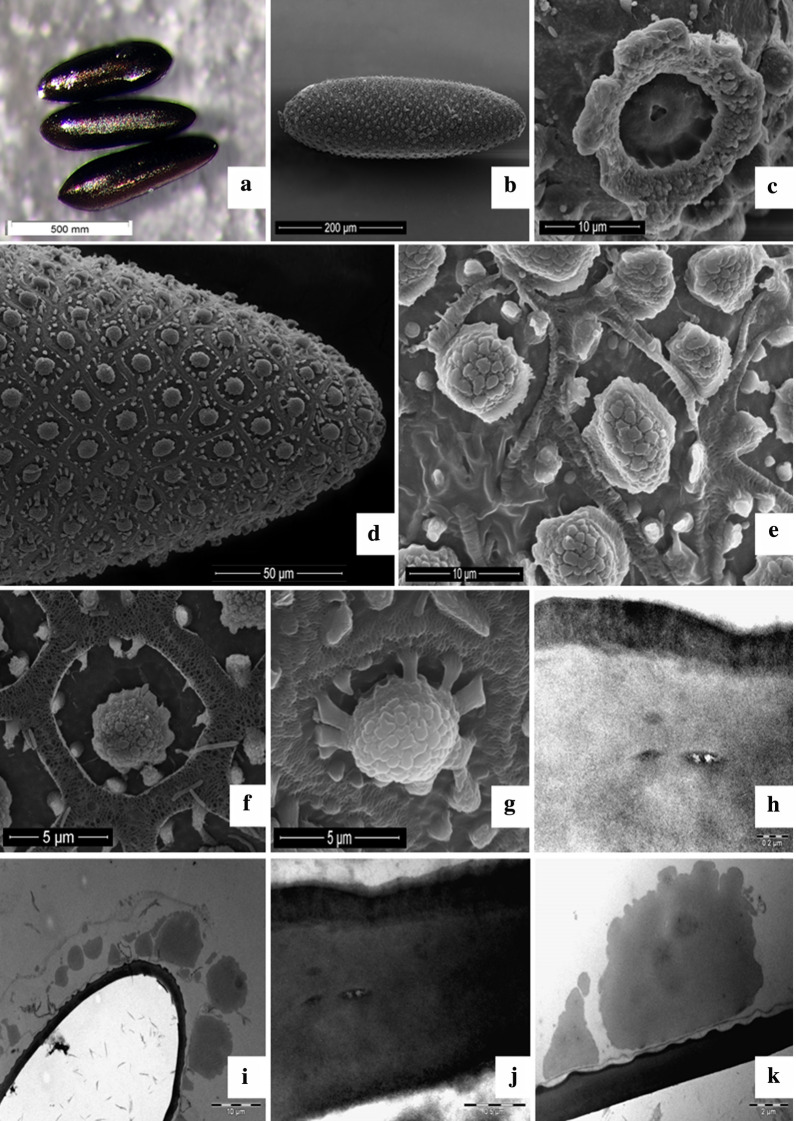
*Aedes aegypti* egg. **a**) Egg with dark, shining aspect measuring less than 1mm and presenting pointy extremities, **b**) mycropylar disc (DM) at the anteroposterior position of the egg, **c**) DM with micropile at the center from where the spermatozoon will fertilize the oocyte, **d**) Posterior extremity pf the egg displaying the ornamentation peculiar to the *Aedes* *aegypti*  egg exochorion,  **e**) Central Tubercle (TC) surrounde by smallers Pheripheral Tubercles (TP), **f**) Pheripheral tubercles (TP) surrounding the Central Tubercle (TC), **g**) Central Tubercle (TC) of the egg, **h**) Exochorion (upper layer) with higher electrodense properties than the  endochorion (bottom layer), **i**) Internal portion of the egg (brighter) in comparison to the external portion (darker layer with TC and TP´s) of the egg that displays the impermeability of the chorion that  hindered the penetration of the resin inside the egg, **j**) Egg chorion  density, **k**) Bigger central tubercle (TC) and smaller peripheral tubercle (TP)

Macroscopically, the eggs of *Ae. aegypti* were whitish at the time of oviposition, and quickly became black. The characteristics identified were a shiny appearance, tapered extremities, bilateral symmetry, and a flattened surface opposite a convex surface (Fig. 1a). Morphometric analysis of the linear dimensions of the population of eggs revealed length of 581.45 ± 39.73 μm and width of 175.36 ± 11.59. The egg index (length/width ratio) was 3.32 ± 0.26 μm. With regard to the measurement of the micropylar (DM) disc diameter of the eggs, it remained at 18.75 ± 1.92 μm (Table 1). The results were also compared with those from the studies by Suman et al. [23], Linley [19], and Faull [24] (Table 2, Fig. 1b).

**Table 1:** Morphometric parameters related to the linear dimension of the population of Aedes aegypti eggs from the Counties of São Paulo- SP and Caxias—MA according to measures of central tendency and dispersion

	Central tendency and dispersion measures				
	Morphometric attributes	Mean	95% Confidence interval	Maximum value	Minimum value
Eggs collected in Sao Paulo	Length	581.45 ± 39.73	569.65–593.25	655.20	521.40
	Width	175.36 ± 11.59	171.92–178.80	199.60	156.50
	Length/width	3.32 ± 0.26	3.24–3.40	4.13	2.78
	Diameter	18.75 ± 1.92	18.18–19.32	22.18	14.27
Eggs collected in Maranhão	Length	580.09 ± 32.30	569.33–590.86	642.40	522.30
	Width	166.75 ± 19.76	160.16–173.34	212.00	135.00
	Length/width	3.51 ± 0.35	3.39–3.65	4.24	2.57
	Diameter	20.79 ± 1.94	20.14–21.43	24.85	17.03

**Table 2:** Comparative analysis between the morphometric findings of *Aedes* *aegypti* eggs identified in this research and the results of Suman et al. [23], Linley [19], and Faull and Williams [24]

	*Ae. aegypti*^a*^	*Ae. aegypti*^b**^	*Ae. aegypti*^c^	*Ae. aegypti*^d^	*Ae. aegypti*^e***^	*Ae. aegypti*^f****^
Length	581.45 ± 39.73	580.09 ± 32.30	625.65 ± 19.91	670.2 ± 7.2	554.41 ± 36.56	562.62 ± 30.85
Width	175.36 ± 11.59	166.75 ± 19.76	183.30 ± 11.04	186.3 ± 2.2	167.65 ± 7.05	160.15 ± 9.73
Length/width	3.32 ± 0.26	3.51 ± 0.35	–	3.61 ± 0.05	3.31 ± 0.18	3.52 ± 0.27
Diameter of micropylar disc	18.75 ± 1.92	20.79 ± 1.94	–	–	33.49 ± 3.9	34.19 ± 5.4

^a^Egg population of São Paulo, ^b^Egg population of Maranhão, ^c^Egg population found by Suman et al [23],  ^d^Egg popultaion found by Linley et al [23], ^e^Egg populations of Cairns-Australia found by Faull and WIlliams [24], **** Egg population of Charters Towers-Australia found by Faull and Williams [24]

Ultrastructurally, SEM showed that the extremities are characterized by poles: The anterior pole, where the entire micropylar apparatus (disc/crown of the micropyle, sectors of the disc of the micropyle and micropyle) is located, is slightly prominent when compared to the opposite pole. The micropylar apparatus maintains a prominent and continuous circular shape (Fig. 1c and d), while the posterior pole is more tapered in relation to the opposite side (Fig. 1c and e). In the external coating of the eggs, regularity was identified with regard to the distribution and shape of the cells, with most maintaining a hexagonal shape (Fig. 1c and e).

In the central region of the chorionic cells, tubers with larger diameters were identified. The tubers were symmetrically arranged, with small peripheral tubers arranged in an organized manner around larger central tubers (Fig. 1f–h). Both appear prominently in the exochorion, thus giving the appearance of high relief or texture.

Complementary transmission electron microscopy (Fig. 1i–l) analysis was used, allowing the identification of ornamentation in the exochorion and confirming that the impermeable characteristic of this structure prevents the entry of fixers and resin inside the cell in the sample processing, causing compromise of these cells. Another finding concerning the exochorion is that it was more electron-dense when compared to its adjacent layer, the endochorion.

### Embryo

**Fig. 2:**
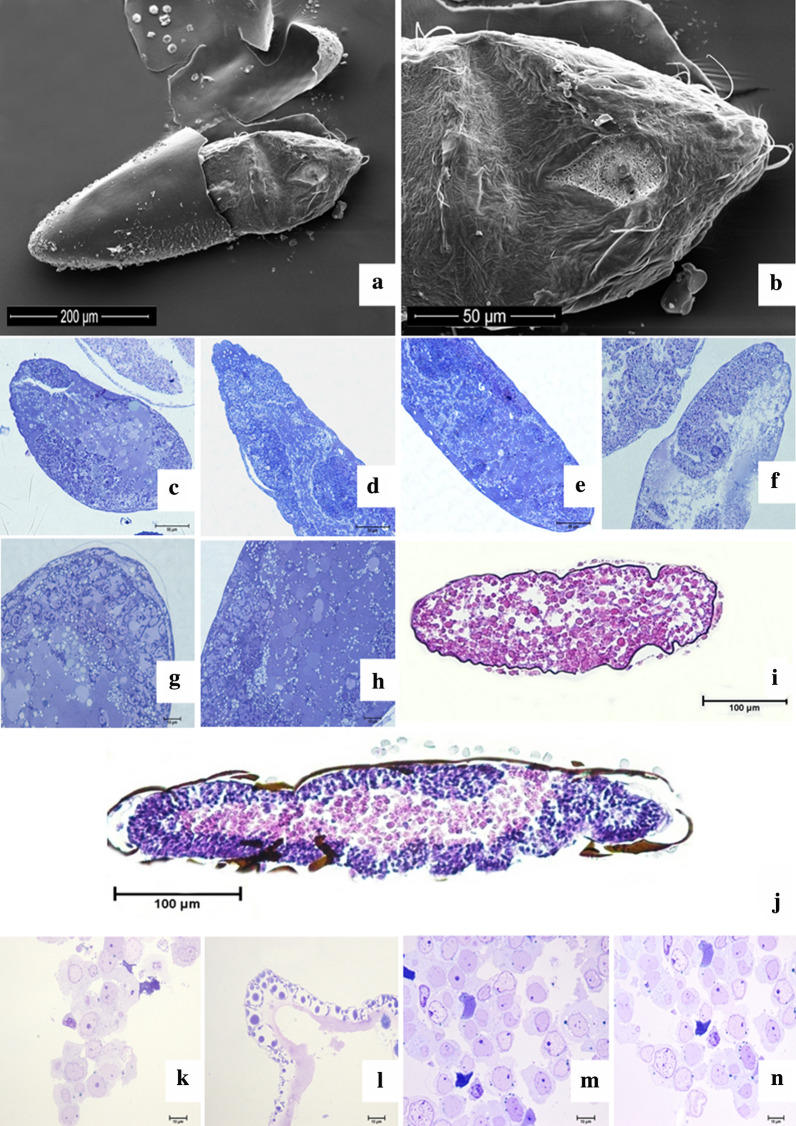
*Aedes aegypti*  initial embyonic development. **a** Sectionated Chorion displaying the initial embryonic structure, **b** Anterior portion of the embryo containing the cephalic structure; **c** Germinal band (discreet embryo folding that outlying the interal layer of the egg), **d** Cranial portion of the embryo, **e** Embryo caudal portion, **f** Distinct embryos sequential development phases, **g** Embryonic cells internally outlying the oocyte, **h** Embryo yelk surrounded by active embryonic cells  **i** Exochorion outlined by the central tubercle and presenting a yelk in the embryo center (initial development), **j** Extension of the germinal band after 18 hours of embryonic development, **k** Embryonic cells at the beginning of embryonic development (5 hours), **l** Eggs exochorion displaying the central and periferical tubercles surronding the egg, **m** and **n**) Embryo cells at the begining of embryonic development (5 hours)

The embryonic development of *Ae. aegypti* occurs inside eggs of up to 1 mm in length (Fig. 1a), which are dark in color and capable of firmly adhering to various substrates. In the period that comprises the initial third of the development of the *Ae. aegypti* embryo, it was possible to verify a scarce presence of bristles in the cephalic region and relatively homogeneous structures in terms of their general morphological aspects. The chorion rupture (Fig. 2a and b) opens the area outside the egg and releases the larva. The correlation between the rupture zone and the micropylar apparatus enables its identification as the cephalic region.

The central (larger) and peripheral tubercles (Fig. 2i and j) were identified as characteristic elements of the exochorion ornamentation in the histological analyses. In the interior of the egg, embryonic cells were spotted, and specifically in the initial third of development (5 h), through the observation of a semi-thin section, round cells with a peripheral nucleus and several cells presenting two nuclei, suggesting cell division (Fig. 2k–n). During the initial stage of embryonic development, 18 h after the analysis of the semi-fine cut, folding of the embryo was found internally to the egg, in about 60% of it a situation called the extension of the germ band. This structure was found during gastrulation, when the ventral blastoderm undergoes extension (Fig. 2c–e), suggesting that the process of intense cellular activity continues after 18 h of the onset of cell development, as a large number of cells were found in the process of division (Fig. 2g and h).

**Fig. 3:**
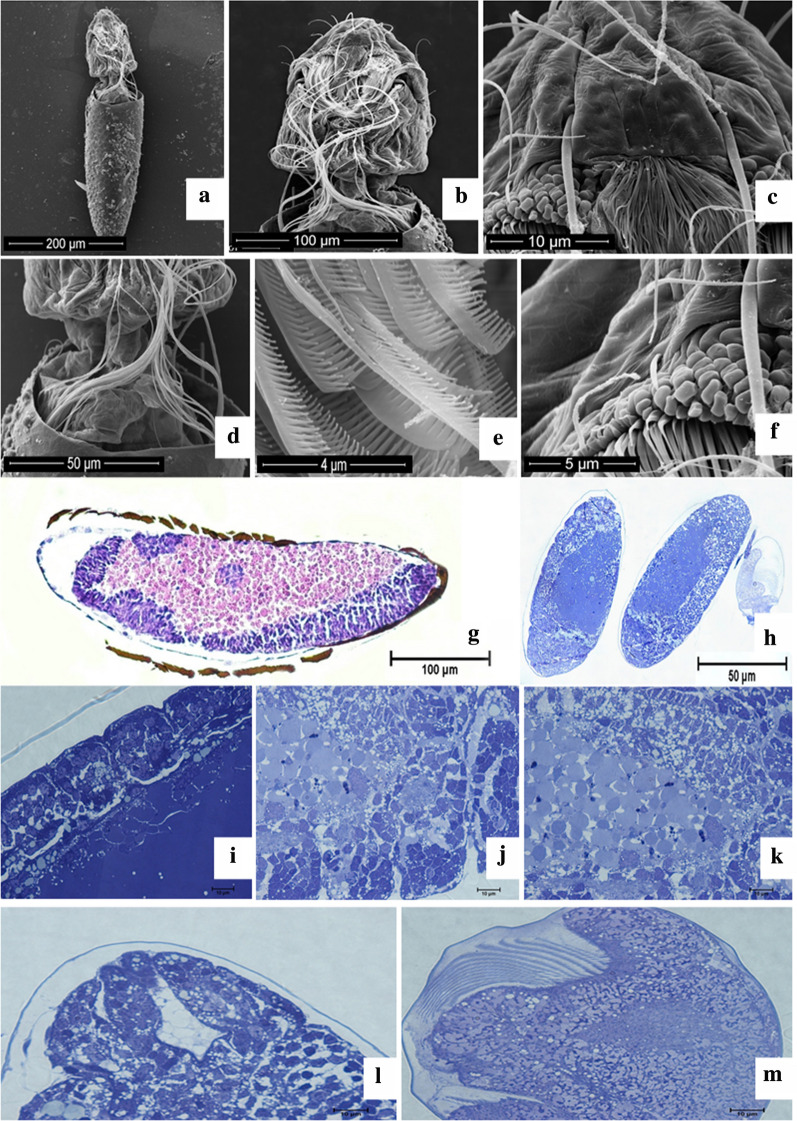
*Aedes* *aegypti* intermediate phase of embryo development.  **a** Sectionated chorion, cephalic and thoracic regions of the embryo, **b** Cephalic portion of the embryo contaning bristles and a naworring gap that divides it from the thoracic region, **c** Anterior extremity of the cephalic region of the embryo, **d** Sectionated chorion in proximitu to the narrowing gap divinding thoracic and cephalic portions, **e** Palatal Brushes, **f** Cells from the cephalic portion, **g** Serous portion in rvidence during the intermidiate phase of embryonic development (25 hours), **h** Embryos after 30 hours of development, **i** and **j** Development of the abdominal portion, **k** Cells with nutritional reserves neighboring the abdominal portion development  **l** Development of the respiratory syphon and caudal appendix, **m** Cephalic portion with bristles and development of the spike at its center

The intermediate phase of development (Fig. 3a–f) is characterized by an increase in the presence of bristles, including palatal brushes, which in the future will allow the larva to perform movements that regulate the acquisition of nutrients through the water flow. There is also a separation of the cephalic and thoracic regions and loss of homogeneity of the general morphological aspect. At this embryonic developmental stage there is an evident membrane surrounding the embryo, and therefore located between it and the chorion, representing the serous layer (Fig. 3g).

During this same stage, embryos were analyzed after 25 h (Fig. 3c) and 30 h of development (Fig. 3h–m). During this period, the abdominal and thoracic segments were established, allowing the emergence of structures such as the respiratory siphon and the last abdominal segment in the form of a caudal appendix (Fig. 3l), a spike that will promote the cleft to release the larva at the time of hatching, in addition to the presence of bristles in the cephalic region (Fig. 3m).

**Fig. 4:**
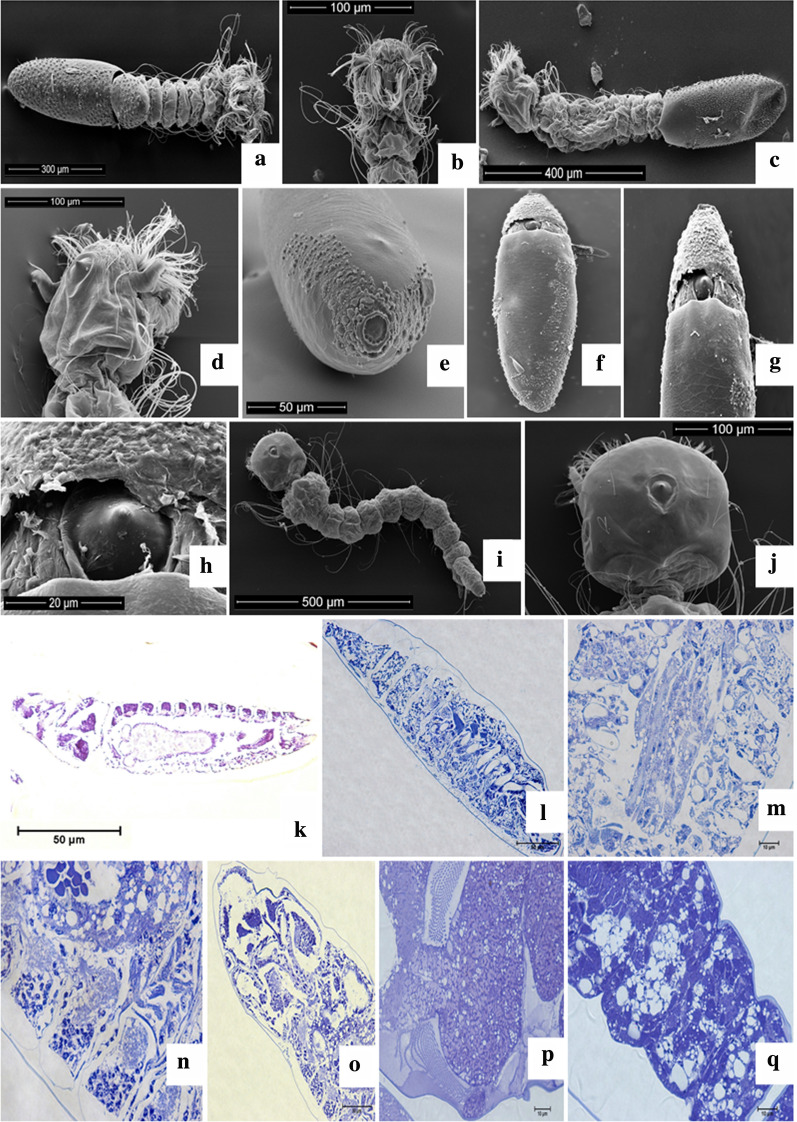
*Aedes* *aegypti *final stage of embryo development. **a** Embryo with fully developed abdominal segments and with the body divide in head, thorax and abdomen, **b** Head and a thorax piece, with briestles in the cephalic region, **c** Embryo with developed abdominal segments, presence of the spike in the dorsal surfice for the egg outbreak, **d** Head and anterior portion of the thorax presenting a feeler, the spike at the center and bristles in the cephalic region; **e** Prominence from where the chorion will be broken, **f** and **g** Evidence of the spike and broken chorion, **h** Zoomed image of the breaking spike, **i** First phase larvae in dorsal vision, **j** Phase 1 larvae head with breaking spike at the center, **k** Embryo during the final phase of development (45 hours), **l** Embryo after 50 hours of development, **m** Fully developed abdominal segments displaying elongated structures composed by diferentiated cells from the insect intestine, **n** Different cells groups and defined abdominal segments, **o** Cephalic and thoracic regions of the embryo after 50 hours of development, **p** Cephalic region with bilateral bristles, **q** Defined abdominal segments

In the final third stage of development (Fig. 4a–d), the body of the future larva already has the divisions in the head, thorax, and abdominal segments, but complete growth is indicated only when the chorion-breaking spike is present (Fig. 4c–h). This structure persists until the first larval stage (Fig. 4i and j), a factor that differentiates that stage from the second larval stage. In the final period of embryogenesis, a prominence in the chorion can be identified (Fig. 4e) in the anterior region, which indicates that the larva is ready to enter the aquatic environment. The prominence site identified was the one that gave rise to the rupture of the transverse fissure in this region by the chorion-breaking spike (Fig. 4f–h) in the egg after the completion of embryonic development. This specific region is where the larva will leave the egg trough. Histologically, the final stage of development (Fig. 4k, l, q) shows that segmentation is better defined, as well as a primitive intestine (Fig. 4m), and the scarce presence of bristles in the cephalic region is replaced by the abundant presence of these structures (Fig. 4p).

**Fig. 5:**
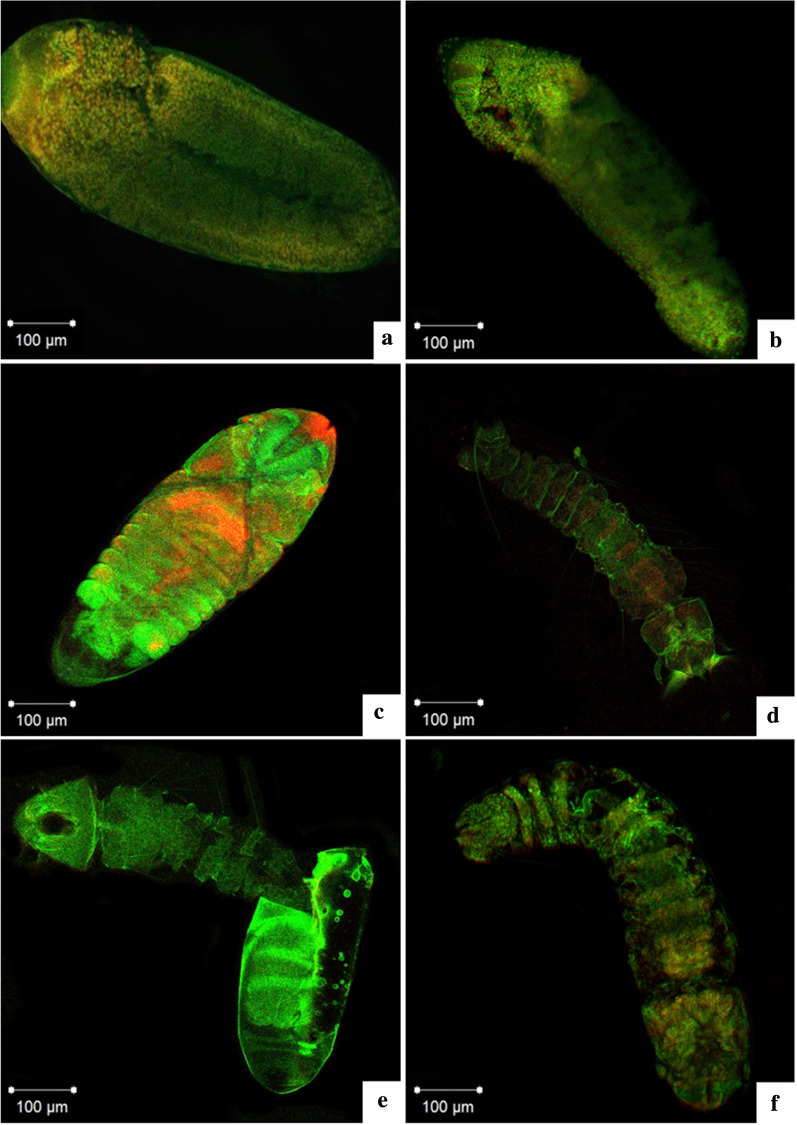
Confocal microscopy of the *Aedes* *aegypti* egg, embryo development and phase 1 larvae. **a** Germinal band extension after 18 hours of embryo development, **b** Embryo after 30 hours developing withcells from the serous layer present in the dorsal surfice, **c** 40 hours of development embryo with dorsal closure and begining of the segmentation of the abdomen, **d** Embryo after 50 hours of embryonic development presenting fully thoracic and abdominal segmentation, **e**) Outbreak of the larvae moment, **f** Diapaused embryo in day 7 with finalized development. It is possible to observe the insect intestine between the sixth and seventh segments of the abdomen

It is known that actin is an important element of the cytoskeleton, and this, in turn, is marked by the reaction with phalloidin, while the nucleus is marked by propidium iodide. The images obtained by confocal microscopy made it possible to follow the sequence of embryonic development after 18 h of development (initial phase) until the moment when the larva was ready to enter the aquatic environment (Fig. 5a–f). In this analysis it was possible to identify the embryo in a situation of extension of the germ band (5B); after 30 h of development the serous cell layer surrounding the embryo (5B) was clear, and at the end of the development at 40 h the process of segmentation and dorsal closure was verified (Fig. 5c), and within 50 h an embryo was observed whose thoracic and abdominal segmentation was well defined (Fig. 5d). It is possible to observe the moment of the larval hatching (Fig. 5e) and the embryo after 7 days diapause, with its development complete, ready to enter the external environment. In this case, the presence of visible segmentation was found, and between the fifth and sixth abdominal segment, it was possible to observe the intestine (Fig. 5f).

Sample fixation for embryo analysis was more difficult than egg morphometric analysis. This was due to the impermeability of the chorion, which made the analyses related to it extremely difficult, needing several repetitions in the different stages of this study. Of approximately 1000 eggs collected, only 46 were successfully processed and generated the results presented.

The samples were fixed in different ways during the study. For analysis under light optical microscopy, 10% formaldehyde solution was used. In transmission electron microscopy they were fixed in 2% glutaraldehyde, and in SEM the biological material was fixed in a modified Karnovsky solution (5% glutaraldehyde and 4% paraformaldehyde in 0.1 M cacodylate buffer, pH 7.2). In optical microscopy with a laser scanning confocal microscope the samples were fixed in 3.7% formaldehyde for 20 min after previous washing, and then subjected to immersion in 3% sodium hypochlorite. Difficulties were identified in all processing techniques, with only 5% of the total samples being completely processed.

## Discussion

The eggs represented by *Ae. aegypti* evaluated in this study were black or brown and measured less than 1.0 mm in length [30, 31]. Even though at the moment of egg-laying they had a white color, they darkened afterward [32]. Other characteristics inherent to the egg were the presence of an oval or elliptical outline, with bilateral symmetry [23, 31–34], all identified in this research.

Fresh eggs are susceptible to water loss, and this condition can impair their viability [36]. This suggests that there would be greater permeability in eggs with shorter embryonic development time, so there would be greater ease in fixing embryonic tissues and obtaining results consistent with the proposed objectives. However, in this research, it was not possible to identify any easier way for processing of the eggs in any of the phases evaluated, with all being equally laborious due to the resistance of the chorion as a constant complicating factor.

The exochorion generally maintains ornamentation that makes it possible to identify the species. It is an excellent parameter for comparing species, as it can reveal significant differences [23, 30, 31, 33, 34]. In Fig. 1c, e–g, the ornamentation on the exochorion maintained the same pattern in the species studied [35]. In addition to *Ae. aegypti*, other culicids of the same genus present ornamentation with polygonal chorionic cells in the exochorion of the egg, where a large central tubercle and other smaller and peripheral ones are observed, except for the anterior surface containing the micropylar apparatus [23, 24]. The central tubercles on the external surface of the egg joined the peripheral tubercles employing thick projections forming a line; however, these differed in terms of their dimensions according to the location in the exochorion [19, 35]. In this study, this projection can be seen in Fig. 1.

With regard to the length of the eggs of *Ae. Aegypti*, the dimensions found in this research were smaller than in the samples collected in India [23]. In another analysis, *Ae. aegypti* from Florida, USA, presented a length of less than 700 µm [19]. The length identified in the egg population of São Paulo also reached lower levels, with a maximum value of 612.39 µm and 621.18 µm, respectively. Regarding the chorionic coating, the findings were similar (Table 2), although there are mathematical differences in their measurements.

The chorion resistance identified is consistent with that previously reported [27], where the precarious description of the embryonic morphology of *Ae. aegypti* was also related to the lack of permeability of the egg. The serous external extraembryonic membrane can be easily seen [35]. This is evident not only in the histological analysis (Fig. 3g), in which the serous layer was between the chorion and embryonic tissues, but also in the analysis using confocal microscopy (Fig. 5b), in which it was characterized as a distinct group of cells when compared to the others. However, in both images, the serosa was more evident in the intermediate phase of embryonic development.

After the completion of the embryonic development, under favorable environmental conditions (clean water, temperature of 27 ± 2 °C, and neutral pH), the eggs of *Ae. aegypti* hatch, with 50% larval hatching [37, 38]. In this study, some eggs with complete embryonic development were analyzed (Fig. 5e and f), and a small proportion of the eggs hatched even after immersion in 3% sodium hypochlorite; that is, even when subjected to unfavorable environmental conditions, approximately 1 h after the beginning of the sample processing, some embryos were still alive, and the eggs hatched.

At the time of hatching, the chorion was ruptured due to the larval muscle activity, which increased in volume and consequently increased the pressure exerted from the spike, a specialized structure; then the rupture of the chorion occurred, from a crack in the part corresponding to the cited coating [32, 39]. This description was verified in this study (Fig. 4e–h), and the spike mentioned is evidenced histologically in Fig. 3m.

## Conclusions

Given the results, it is possible to surmise that there is a greater resistance of the chorion in the embryo, a factor that complicates the microscopic analysis of the embryo (mainly due to fixation and processing failure), as well as a strong protective barrier, making it difficult to use these cells in studies. No weaknesses were identified in the egg phase of the biological cycle, which is extremely important for further research to identify new ways to effectively combat this important vector.

## Data Availability

The data will be available upon request.
